# Development and Characterization of Monoclonal Antibodies for the Detection of Fish Protein

**DOI:** 10.3390/foods10102360

**Published:** 2021-10-04

**Authors:** Yi-Tien Chen, Yun-Hwa Peggy Hsieh

**Affiliations:** 1 School of Food Safety, Taipei Medical University, Taipei City 110, Taiwan; ytc@tmu.edu.tw; 2 Department of Nutrition and Integrative Physiology, Florida State University, Tallahassee, FL 32306, USA

**Keywords:** fish allergy, monoclonal antibody, muscle protein, thermal-stability, immunoassay, detection

## Abstract

This study developed and characterized anti-fish monoclonal antibodies (mAbs) capable of detecting fish, a major allergenic food, in processed food products to protect fish sensitized individuals. Of the three mAbs raised against crude protein extract of cooked fish muscle, mAb 8F5 exhibited a positive reaction to all 50 common food fish species tested with no cross-reactions to shellfish, land animals, or food additives. Although the ELISA results were negative against swordfish and yellowfin tuna, western blot clearly detected both after cooking. The ~36 kDa antigenic protein of mAb 8F5, which was found in all fish species, was detectable by mAb 8F5 in all of the fish samples even after prolonged heat treatment (100 °C, up to 60 min). These findings suggest that mAb 8F5 has great potential utility as a probe for the immunochemical detection of fish tissue in cooked food.

## 1. Introduction

Fish is classified as one of the eight major allergenic foods (fish, shellfish, peanut, soybean, wheat, tree nuts, milk, egg) under the Food Allergen Labeling and Consumer Protection Act (FALCPA) [1], which requires accurate information on food allergens to be included in the label for food products. Labeling fish as a food allergen on food packaging is mandatory in many countries. Surveys suggest that 0.9% of the population suffers from a fish allergy in the United States, and 0.7% in European countries [2,3]. About 45% of children with fish allergy outgrows it in adolescents [4]. However, there is no medical cure for food allergy. Sufferers must therefore strictly avoid any food containing fish to reduce the risk of a severe allergic reaction.

To protect fish-allergic patients, a number of assays have been developed to detect the presence of fish tissue in foods, most of which are protein-based immunoassays or DNA-based polymerase chain reaction (PCR) techniques. While PCR methods require trained personnel and instruments to perform the test and evaluate the data, immunoassays based on highly specific antibody–antigen interactions can be developed into simple and rapid assays such as one-step lateral flow strip tests. Immunoassays are now widely used in clinical, food, industrial, and biological applications targeting specific substances. Parvalbumin, which is generally recognized as the major allergen in fish [5,6,7], is often used as the marker protein to indicate the presence of fish in foods. Previous reports on the immunoassays developed for this purpose mainly utilize polyclonal antibodies (pAbs) raised against fish parvalbumin. Fæste and Plassen [8] developed a pAb based sandwich ELISA for the detection of fish parvalbumin; however, the assay did not detect all 32 fish samples tested. Shibahara et al. [9] also developed anti-fish parvalbumin pAb, which reacted to sample extracts of all 22 fish species tested, but cross-reacted to cephalopod samples. Their sample extracts were either prepared from heated protein extracts from raw fish samples or from fish powders, so the ability of these assays to detect cooked fish was not verified, even though cooked fish and its products are by far the most common for human consumption. A monoclonal antibody (mAb) 3E1 and the antibodies developed by phage display technology have also been developed for the detection of fish parvalbumin [10,11]. mAb 3E1 was unable to detect certain fish-species (e.g., pollock, yellowfin tuna and salmon) [10]. The antibodies constructed by phage display were not applied to detect any fish samples [11]. In addition, the amount and thermal stability of parvalbumin varies among fish species [12]. Therefore, those antibodies against parvalbumin do not provide reliable performance in the detection of fish in food products.

Detection methods targeted other fish allergens such as fish collagen [13] and multiple enzymes including creatine kinase (41 kDa) [14], triosephosphate isomerase (26 kDa) [15], glyceraldehyde-phosphate dehydrogenase (38 kDa) [15], enolase (50 kDa) [16], and aldolase (40 kDa) [16] have not been developed to detect the presence of fish tissues in cooked food. Fish collagen degraded after heating and then became weak in allergenicity [13] and these enzymes are not thermally stable, so lose allergenicity after heat treatment. Tropomyosin (36 kDa), a thermal stable muscle protein, was first identified as an allergen in tilapia [17] and later in cod, albacore, and swordfish [18]. However, three commercial fish immunoassay kits against fish parvalbumin or tropomyosin could only achieve a detection rate for 57 bony fish from 26% to 61% [19].

In an earlier study, we developed a sandwich enzyme-linked immunosorbent assay (sELISA) using pAbs raised against a mixture of crude protein extracts of cooked fish muscle from ten species (Atlantic salmon, yellowfin tuna, swordfish, black grouper, tilapia, red snapper, amberjack, basa, catfish, and perch) [20]. After cleaning by immunoabsorption, the pAbs recognized a major 36 kDa protein band in the sample protein extract. This pAb-based sELISA was found to be capable of recognizing all 63 fish species tested, with no cross-reactions with land animals, poultry, or common food additives. Unfortunately, although pAbs are relatively easy and more economic to develop, their specificity and quality are inherently inconsistent from batch to batch. In contrast, a monoclonal antibody (mAb) produced from a single clone of a hybridoma cell has clearly defined characteristics and recognizes only a specific epitope on the antigenic protein. Therefore, in order to provide an effective mAb-based immunochemical method for fish detection, the specific objectives of the present study were to (1) develop fish-specific mAbs raised against crude protein extracts from cooked fish; (2) characterize these mAbs including their selectivity, isotypes, and antigenic proteins; and (3) demonstrate the usefulness of a selected mAb as a probe in an immunoassay for the detection of fish even after heat treatment.

## 2. Materials and Methods

### 2.1. Samples

The fifty species of fresh or frozen fish samples used in the study were either provided by the State of Florida’s Department of Agriculture and Consumer Services or purchased from a reputable seafood distributor. Effort was made to collect as many commonly consumed food fish species as possible including a wide variety of fish species either from domestic waters or of imported commodities. The three shellfish and eleven land animal meat samples were purchased from local food stores. All samples (listed in Table 1) were stored at −80 °C until use.

### 2.2. Preparation of Protein Extracts

To prepare protein extracts of the samples, lean muscle tissue from fresh fish, shellfish and land animals of each species was minced individually without cross contamination, and a 5 g portion of each sample was weighed into a beaker. For the cooked samples, a beaker containing minced raw muscle tissue was covered by aluminum foil and heated in a boiling water bath for 8 min. Twenty five mL of 0.15 M NaCl solution was then added to the beakers containing either raw or cooked fish muscle tissue, after which each sample was homogenized for 1 min at 13,000 rpm using an ULTRA-TURRAX T25 basic homogenizer (IKA Works, Wilmington, NC, USA), held at 4 °C for 2 h and then centrifuged (10,000× *g* at 4 °C, 30 min) and filtered through a Whatman No. 4 filter paper (Whatman, Piscataway, NJ, USA). The clear protein filtrates were stored in small vials at −20 °C until use. The extracts of four common food additives (porcine gelatin, egg albumin, non-fat dried milk, and soy meal) were prepared as 1% (*w/v*) solutions in phosphate buffered saline (PBS) and then extracted in the same manner as the muscle samples. Protein concentrations for each extract were determined using a Protein Assay Kit II (Bio-Rad) according to the manufacturer’s protocol using bovine serum albumin (BSA) as the standard. The extracts were stored at −20 °C until use.

### 2.3. Fish Sample Preparation for Studying the Effect of Heating Times

To examine the effect of heating time on the immunoreactivity of the mAb with different fish, fresh muscle tissue from three fish species (swordfish, yellowfin tuna, and cod) was cut into cubes (1.5 cm^3^) of uniform shape and thickness. These pieces were placed in individual small beakers covered with aluminum foil and heated in a boiling water bath for different lengths of time [0 min (raw), 1 min, 3 min, 5 min, 10 min, and 15 min]. The protein concentrations of the protein extracts were determined using a Protein Assay Kit II (Bio-Rad). To investigate the thermal stability of the antigen–antibody binding, muscle tissue cubes of fresh cod were heated in a boiling water bath for different lengths of time [0 min (raw), 5 min, 10 min, 15 min, 20 min, 30 min, 40 min, 50 min, and 60 min]. After cooling, five vol. (*v/w*) of 0.15 M NaCl solution was added to each and their protein extracts prepared as described above.

### 2.4. Development of mAbs

As the main objective of this study was to develop mAbs that specifically targeted only thermal stable fish antigenic proteins, the immunogen was prepared from a crude protein extract of cooked red snapper following the same procedure as that described for the protein samples in the previous section. The cooked fish protein extract was dialyzed (M.W. cut-off of 10 kDa) in 10 mM PBS for 24 h and then filtered through a 0.2 μm filter. The clear protein filtrate was emulsified with Freund’s complete adjuvant as the immunogen. The immunization and the following hybridoma procedures were performed in the Hybridoma Facility at Auburn University, Auburn Alabama in compliance with the University’s Animal Welfare guidelines. The details of the subsequent procedures used for the immunization, boosting, hybridoma production, screening, and cloning were as described in [10]. Based on the preliminary screening data, three clones were selected for further study. The Pierce^®^ Rapid ELISA Mouse mAb Isotyping Kit (Thermo Fisher Scientific, USA) was used to determine the isotypes of the selected mAbs and performed according to the protocol specified by the manufacturer. Properly diluted supernatants were used in this study.

### 2.5. Indirect Enzyme-Linked Immunosorbent Assay (iELISA)

iELISA was used to examine the titers of the mouse sera, positivity of the hybridomas, and the species selectivity of the three selected mAbs. The immunogen and the individual fish protein extracts from 50 fish species, 11 land animal species, three shellfish species, and four food additives (Table 1), together with positive (cooked red snapper extract) and negative (BSA) controls, were each diluted appropriately in 0.06 M carbonate buffer and then 2 μg per well coated onto a microplate and held at 37 °C for 1 h. The wells were washed three times by an ImmunoWash microplate washer (Bio-Rad, Hercules, CA, USA) with PBS containing 0.05% Tween 20 (PBST) and blocked with blocking solution (1% BSA in PBS) at 37 °C for 1 h. The 100 μL diluted supernatants (1:5 in PBST with 1% BSA) of the mAbs were added to the wells and the plate was incubated at 37 °C for 1h. After a further washing step, 100 μL diluted (1:3000) horseradish peroxidase (HRP)-conjugated goat anti-mouse immunoglobulin (Ig) G (Sigma-Aldrich, St. Louis, MO, USA) was added and the plate incubated at 37 °C for another 1 h. The plate was then washed three times and 100 μL 2,2-azino-di-[3-ehyl-benothiazoline-6-sulfonic acid] (ABTS) solution added to develop the color at room temperature (RT) for 30 min. The color development was stopped by adding 100 μL stop solution (0.2 M citric acid) per well. The absorbance was measured at 415 nm using a PowerWave X microplate reader (BioTex, Winooski, VT, USA). The absorbance values for the iELISA were calculated by analyzing two replicates of each sample.

### 2.6. Sodium Dodecyl Sulfate Polyacrylamide Gel Electrophoresis (SDS-PAGE) and Western Blot (WB)

SDS-PAGE was performed according to the standard procedure [21] using 4% SDS-polyacrylamide stacking gel with 12% SDS-polyacrylamide separating gel. The sample extracts were mixed with sample buffer (working concentration: 62.5mM Tris-HCl, 2% SDS, 10% glycerol, 0.05% 2-mercaptoethanol, 0.002 % bromophenol blue) and heated in a boiling water bath for 5 min. To characterize the antigenic protein, a fixed volume of protein per lane of raw and cooked fish protein extract was loaded onto the gel. The electrophoresis was performed at 100 V for 120 min using Mini-PROTEAN II electrophoresis (Bio-Rad). The polyacrylamide gels were stained with EZ Blue^™^ Gel staining (Sigma-Aldrich) according to the manufacturer’s instructions.

After the SDS-PAGE was completed, the separated protein bands on the other gel set were transferred onto a nitrocellulose membrane (Bio-Rad) at 300 mA for 1 h using Mini Trans-Blot (Bio-Rad) with the transfer buffer (Bio-Rad). The membrane was blocked with 1% BSA in Tris-buffered saline (TBS). After washing in TBS with 0.05% Tween-20 (TBST), the membrane was blotted by incubating with the mAbs supernatant diluted 1:5 in TBST with 1% BSA (antibody buffer) for 1 h at RT. The excess antibody solution was washed away by TBST and the membrane incubated with goat anti-mouse IgG alkaline phosphatase conjugated antibody (Bio-Rad) diluted 1:3000 in the antibody buffer for 1 h at RT. The antigenic proteins appeared as dark purple bands after incubating the membrane with BCIP/NBT (Thermo Fisher Scientific) solution for 3 min.

## 3. Results and Discussion

### 3.1. Species Selectivity of the Newly Developed mAbs

A panel of three hybridomas, 2A4, 3F5, and 8F5, was initially selected and cloned based on their strong reactivity with the immunogen. The isotypes of 2A4, 3F5, and 8F5 were IgG1, IgG2b, and IgG2a, respectively. After expanding the clones, the species selectivity of each was examined against cooked sample extracts of 50 common food fish species, three shellfish, and 11 land animals (Table 1) using iELISA. The overall results are summarized in Table 2. All three of these mAbs showed strong immunoreactivity with most or all of the fish species tested. Although mAb 2A4 and 3F5 did react to cooked samples of all 50 fish species, both also cross reacted with most of the land animal samples, indicating that the epitopes of mAb 3F5 and 2A4 on their antigenic proteins are located in a conserved region of both fish and land animal species. In contrast, mAb 8F5 reacted strongly with 48 of the 50 cooked fish samples with no cross-reaction with any of the non-fish samples including commonly consumed shellfish, land animals, and food additives. The iELISA results (Table 1) indicate that mAb 8F5 recognizes a conserved region on the amino acid sequence of the antigenic protein that is unique to fish and is not found in any of the representative non-fish species tested.

The iELISA absorbance threshold for a qualitative determination of the positive/negative results in this study was set at OD = 0.2; this value is widely used as the cut-off by many analytical, biological, and medical researchers [22,23]. An OD value below 0.2 is the detection limit for visual inspection and is therefore generally recognized as negative in commercial immunoassay kits. In this study, only two fish samples, swordfish and yellowfin tuna, produced very weak reaction signals with mAb 8F5 and were thus initially classed as negative samples. Further investigations explored the reasons for this weak or lack of reaction of these two species with mAb 8F5.

### 3.2. Antigenic Components Recognized by the Newly Developed mAbs

**Figure 1:**
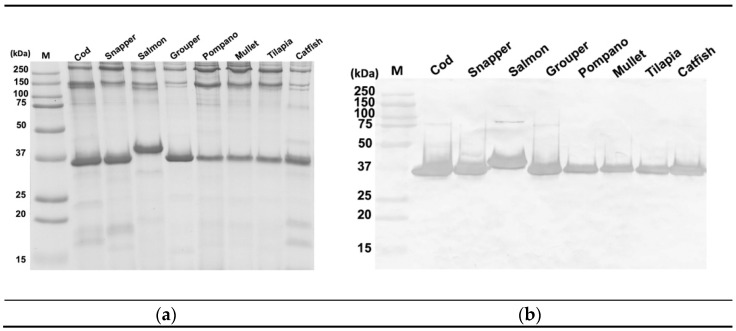
SDS-PAGE (**a**) and western blot analysis (**b**) of cooked fish samples using mAb 8F5 supernatant (1:5). The amount of sample loaded on the 12% SDS-PAGE was 6 μL per lane. M: molecular weight marker.

Randomly selected fish species from positively reacting fish samples were used to examine the antigenic protein components of the three new mAbs, 2A4, 3F5, and 8F5 by WB. Interestingly, this revealed that all three mAbs recognized a 36 kDa antigenic protein band (Table 2 and Figure S1). Due to the substantial cross-reactivity with land animal proteins exhibited by mAbs 2A4 and 3F5, they are not useful for specific fish detection. Therefore, only the fish-specific mAb 8F5 was selected for further investigation in this study. While WB results for mAb 2A4 and 3F5 are attached in the Supplementary Materials as Figure S1, the SDS-PAGE protein profile for the extracts of the eight species (cod, snapper, salmon, grouper, pompano, mullet, tilapia, and catfish) of cooked fish (100 °C for 8 min) and the antigenic protein bands revealed by WB using mAb 8F5 are shown in Figure 1.

WB revealed that this 36 kDa protein is the antigenic protein consistently recognized by mAb 8F5 in all of these fish species (Figure 1b), although the molecular weight of this antigenic protein in salmon is slightly heavier than that in the other species. These results confirm that the 36 kDa protein is the major thermal stable protein appearing in all the fish samples, and it is also the antigen for the mAb 8F5. These suggest that this mAb recognizes a unique fish conserved region on the protein sequence of this 36 kDa protein. The pAb reagent used in the previously reported sELISA, which successfully detected all 63 fish species tested, also recognized a major 36 kDa protein band in all the cooked fish samples [20]. The 36 kDa protein recognized by mAb 8F5 and pAb could be the same antigenic protein because their immunogens were similarly prepared in our laboratory.

### 3.3. Antigenic Protein Profile of Yellowfin Tuna and Swordfish

The 8 min cooked yellowfin tuna and swordfish exhibited either very weak or negative immunoreactivity (OD < 0.2) with mAb 8F5 in iELISA (Table 1). In order to investigate and ensure that mAb 8F5 has the capability to detect all the fish species tested, the immunoreactivity of yellowfin tuna and swordfish were further studied using WB to examine whether heat treatment affected their antigen exposure, thus the immunoreactivity against mAb 8F5. As noticed during the protein extraction step performed for this analysis, the extracted protein concentration of these two fish species decreased much faster after heating compared with the other fish samples, which is why the soluble protein concentrations extracted from these two fish species heated for shorter times (0 min, 1 min, 3 min, 5 min, 10 min, and 15 min) than the other samples were examined (Table 3). A cod sample was included in the analysis as the positive control for comparison. The results show that the soluble protein concentrations in the extracts of raw swordfish and yellowfin tuna were 8.73 mg/mL and 16.09 mg/mL, 2.3 to 4.2 times higher, respectively, than that of raw cod (3.83 mg/mL). The protein concentration of all three fish samples decreased dramatically after just 1 min to 5 min of heating. After 15 min of heating, the protein concentrations of swordfish (0.31 mg/mL) and yellowfin tuna (0.29 mg/mL) were far below that of cod (1.17 mg/mL), indicating that the muscle protein of these two species is considerably more heat-labile than the other mAb 8F5 positive species such as cod. The thermal stability of muscle protein is thought to be different for different fish species due to the ambient temperature of their habitat and the style of swimming the fish typically engage in [24,25,26]. The unusual characteristics in the heat stability of the muscle proteins of yellowfin tuna and swordfish may be due to the way they move between different ambient temperature zones and their particular exercise style.

**Figure 2:**
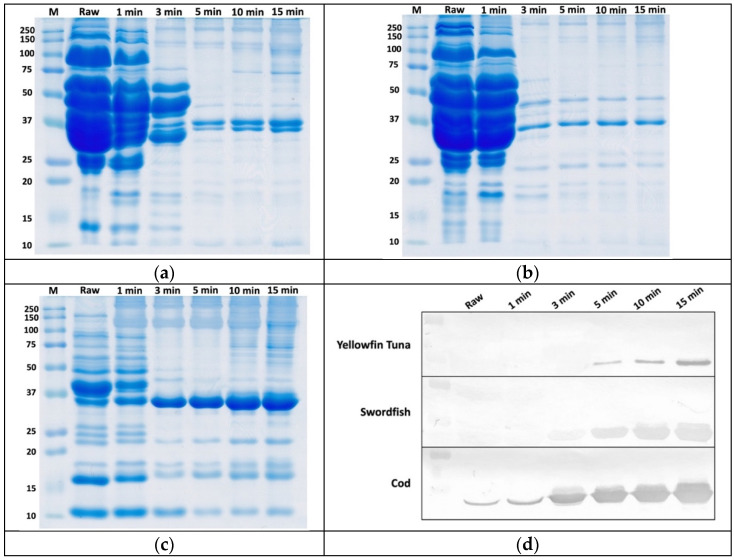
SDS-PAGE gel images (**a**–**c**) and western blot antigenic protein profiles using mAb 8F5 supernatant (1:5), (**d**) of yellowfin tuna, swordfish, and cod cooked for different lengths of time. The sample loading was 6 μL per lane. M: Molecular weight marker.

We then examined the effect of heating time (from 0 to 15 min) on the immunoreactivity of the antigenic protein with mAb 8F5 using WB. The SDS-PAGE images (Figure 2a–c) and the combined WB results (Figure 2d) closely matched the observations regarding the difference in the amount of the total soluble proteins and the 36 kDa antigenic protein, respectively, in different fish species with different heating periods. In SDS-PAGE gels, multiple protein bands appeared in raw and 1-min cooked samples, but gradually decreased to only a few bands after 5 min of heating (Figure 2a–c). The 36 kDa band seems to appear in the SDS-PAGE gels of both raw and cooked fish samples. No or very weak antigenic bands were observed in yellowfin tuna and swordfish in raw and cooked fish below five minutes, however, the 36 kDa protein band appeared from 5-min of cooking onward. Compared to the cod samples (cooked for 5 min to 15 min), the antigenic protein bands of yellowfin tuna and swordfish were much weaker. This experiment loaded a fixed volume (6 μL per lane) of protein extracts on the gel to examine any changes in the amount of the antigen in the fish extracts for different heating times. Even though these two species contain much higher amounts of total soluble proteins than cod in raw tissue, heating quickly denatures and insolubilizes most of these proteins and the thermal stable 36 kDa protein remains in the extract at an increased proportion related to the other proteins. For less than 15 min of cooking time, the longer the heating, the stronger the antigenic band appeared in all three species, suggesting more hidden epitopes were gradually exposed as this peptide chain unfolded upon heating.

Although yellowfin tuna and swordfish produced negative iELISA results, WB revealed that mAb 8F5 does indeed react with these two fish species, targeting the same antigen. The weak iELISA signals for yellowfin tuna and swordfish are likely due to the low antigen concentration in their muscles and the relatively small amount of protein adsorbed on each well. A higher concentration with longer heat treatment is required to reveal the immunoreactivity of this group of fish species.

### 3.4. Thermal Stability of the mAb 8F5 Epitope

**Figure 3:**
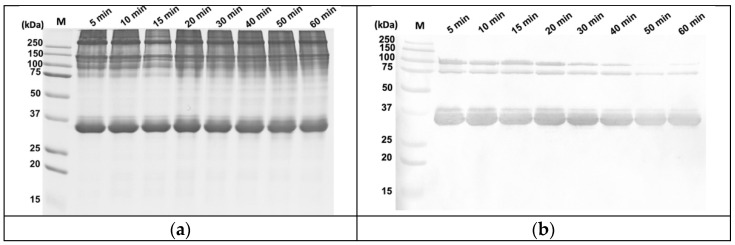
SDS-PAGE (**a**) and western blot analysis (**b**) of cooked cod treated for different heating times using mAb 8F5 supernatant (1:5). The amount of sample loaded on the 12% SDS-PAGE was 15 μL per lane. M: molecular weight marker.

Heating is the most commonly used processing method for fish before consumption. Most muscle proteins are sensitive to heat and become denatured after heating. The configuration of the epitope on the antigenic protein can be impeded by heating, affecting the immunoreactivity due to the obstructed antibody–antigen binding [27,28,29]. The thermal stability of the epitope is thus a critical factor for the immunoreactivity and, hence, the detectability of the antigenic protein. To ensure detectability in fish samples that have undergone lengthy heating times, we also investigated the effect of heating time (up to 60 min) on the thermal stability of the epitope on the 36 kDa antigenic protein with mAb 8F5. Cod was selected as a representative species because it exhibited similarly strong iELISA signals as the majority of other positive species tested and is one of the top ten seafood species consumed worldwide [30]. The SDS-PAGE and WB results are presented in Figure 3a,b, respectively. More proteins (15 μL) were loaded on each lane of the gel in this experiment than the previous one (6 μL) in order to observe the change in immunoreactivity of the fish over this wide range of heating times (from 5 to 60 min), which is why the overall color intensity of the protein bands in this figure appeared darker than those shown in Figure 1 and Figure 2. The SDS-PAGE profile revealed that the 36 kDa antigenic protein did indeed retain its integrity for up to 60 min of heating time, with only a slight decrease in the color intensity of the bands after 30 min of heating (Figure 3a). The WB results showed a total of three antigenic bands on the membrane; in addition to the main 36 kDa protein, there were two minor protein bands at around 75 kDa and 100 kDa. The intensities of all three decreased after 30 min of heating (Figure 3b), indicating that excessive or prolonged heating gradually and damages the epitope to some extent. It is likely that the 75 kDa and 100 kDa bands are the dimers and trimers of the 36 kDa antigenic protein because they also bind to mAb 8F5. These results suggest that the epitope of mAb 8F5 is highly thermally stable even after prolonged heating in boiling water for up to 60 min, suggesting that mAb 8F5 can be used for the detection of fish in products that have been cooked for a wide range of cooking times.

The pAb previously used in the sELISA apparently recognized the same major 36 kDa protein in all 63 cooked fish samples tested [20]. This supports our contention that this protein is not only highly antigenic, but also ubiquitously present in all common food fish species with fish-specific epitopes, making it an ideal biomarker for the immunodetection of fish. Therefore, it is important to further investigate the identity of the 36 kDa protein. Based on its thermal stability and molecular weight, the 36 kDa antigen is likely to be fish tropomyosin. The identification and characterization of this 36 kDa protein has been performed and the findings will be reported separately.

## 4. Conclusions

In this study, we developed a fish-specific mAb, 8F5, that cross-reacts with all 50 common food fish species tested without exhibiting any cross-reactions with the 18 shellfish, land animals, or food additives tested. The 36 kDa antigenic protein of mAb 8F5 that appears in both raw and cooked fish of all species is a thermally stable muscle protein common to all the fish species tested; the binding was thermally stable up to 60 min of heat treatment at 100 °C. Characterization of this 36 kDa protein is of great interest and importance as it has the potential to serve as an excellent fish marker protein. The high thermal stability and fish-selectivity of the epitope recognized by mAb 8F5 demonstrate the potential utility of this mAb in the future development of various forms of immunoassays for the specific detection of fish in cooked foods. The real utility must be assured by a complete validation, performed with the analysis of real commercial and complex food samples, especially, those indicating in the label that “may contain” or do-not-contain fish, where the concentration of this protein can be very low or is being hidden by the food matrix, which would really assure consumer protection.

## Figures and Tables

**Table 1:** Immunoreactivity of 3 anti-fish mAbs (8F5, 2A4, 3F5) against 50 species of fish, three shellfish, 11 land animals, and four food additives by indirect ELISA.

Market Name	Scientific Name	Immunoreactivity ^a^		
Fish Species		mAb 8F5	mAb 2A4	mAb 3F5
Cod	*Gadus Morhua*	+++	++	+++
Orange Roughy	*Hoplostethus Atlanticus*	+++	++	++
Striped Bass	*Morone Saxatilis*	+++	++	+++
Gray Snapper	*Lutjanus Griseus*	+++	++	+++
Black Sea Bass	*Centropristis Striata*	+++	++	++
Spotted Seatrout	*Cynoscion Nebulosus*	+++	++	+++
Lane Snapper	*Lutjanus Synagris*	+++	++	+++
Mahi-Mahi	*Coryphaena Hippurus*	+++	++	++
Cubera Snapper	*Lutjanus Cyanopterus*	+++	++	+++
Sheephead	*Archosargus Probatocephalus*	+++	++	+++
Vermilion Snapper	*Rhomboplites Aurorubens*	+++	++	+++
Yellowtail Snapper	*Ocyurus Chrysurus*	+++	++	+++
Tra	*Pangasius Hypothalmus*	++	++	++
Red Grouper	*Epinephelus Morio*	+++	++	+++
Gag Grouper	*Mycteroperca Microlepis*	+++	++	++
Tomato Hind	*Serranus sonnerati*	+++	+++	+++
Orange Spotted Grouper	*Epinephelus coioides*	+++	++	+++
Atlantic Salmon	*Salmo Salar*	++	++	++
Southern Flounder	*Paralichthys Lethostigma*	+++	++	+++
Cobia	*Rachycentron Canadum*	+++	++	+++
Black Grouper	*Mycteroperca Bonaci*	+++	++	+++
Scamp Grouper	*Mycteroperca Phenax*	+++	++	+++
Wahoo	*Acanthocybium Solandri*	+	++	++
Haddock	*Melanogrammus Aeglefinus*	+++	++	+++
Pollock	*P. Pollachius*	++	++	++
Hog Snapper	*Lachnolaimus Maximus*	+++	++	+++
Tilapia	*Oreochromis Niloticus*	+++	++	+++
Red Snapper	*Lutjanus Campechanus*	+++	++	++
Pompano	*Trachinotus Carolinus*	+++	++	+++
Mullet	*Mugil Gyrans*	+++	++	+++
Yellow Edge Grouper	*Variola Louti*	+++	++	+++
Alaskan Halibut	*Hippoglossus Stenolepsis*	+++	++	+++
Rainbow Trout	*Oncorhynchus mykiss*	+++	++	+++
Catfish	*Ictalurus punctatus*	+++	++	+++
Bluegill	*Lepomis macrochirus*	+++	++	+++
Chinook salmon	*Oncorhynchus tshawytscha*	+++	++	+++
Ocean Perch	*Sebastes alutus*	+++	++	+++
Mangrove Snapper	*Lutjanus griseus*	++	++	+++
Whiting	*Menticirrhus littoralis*	++	++	++
Basa	*Pangasius bocourti,*	+++	+++	+++
Camouflage Grouper	*Epinephelus polyphekadion*	+++	+++	+++
Coral Trout	*Plectropomus leopardus*	+++	+++	+++
Dusky Grouper	*Epinephelus marginatus*	+++	+++	+++
Redmouth Grouper	*Aethaloperca rogaa*	+++	+++	+++
Squaretail Grouper	*Plectropomus areolatus*	+++	++	+++
Trout Cod	*Maccullochella macquariensis*	+++	+++	+++
Wavy Lined Grouper	*Epinephelus undulosus*	+++	++	+++
Caribbean Red Snapper	*Lutjanus purpureus*	+++	++	++
Yellowfin Tuna	*Thunnus Albacares*	-	++	++
Swordfish	*Xiphias Gladius*	-	+	+
**Non-Fish Species**				
White Shrimp	*Litopenaeus setiferus*	-	-	-
Blue Crab	*Callinectes sapidus*	-	-	-
Scallop	*Pectinidae*	-	-	-
Chicken	*Gallus Domesticus*	-	+	-
Turkey	*Meleagris*	-	++	++
Pork	*Sus Scrofa Domesticus*	-	++	++
Beef	*Bos Primigenius*	-	++	++
Lamb	*Ovis Aries*	-	+	-
Rabbit	*Oryctolagus Cuniculus*	-	++	++
Horse	*Equus Ferus Caballus*	-	++	++
Deer	*Cervidae*	-	++	++
Elk	*Cervus canadensis*	-	++	++
Rat	*Rattus*	-	++	+++
Frog	*Lithobates catesbeianus*	-	++	+++
**Food Additives**				
Gelatin		-	-	-
Egg albumin		-	-	-
Soy protein		-	-	-
Nonfat Dried Milk		-	-	-

^a^ Immunoreactivity was examined by iELISA using the 1:5 diluted supernatants of mAbs (8F5, 2A4, 3F5). Samples were coated at 2 μg/well in microplates. Absorbance readings over 0.2 were considered “positive”; those less than 0.2 were considered negative. The absorbance reading: <0.2: “-”; 0.2–0.5: “+”; 0.5–1.5: “++”; ≥1.5:”+++”.

**Table 2:** Isotypes, antigenic proteins, and summarized iELISA results against 50 species of fish, three shellfish, 11 land animals, and four food additives of the three anti-fish mAbs.

			Immunoreactivity against							
mAb	Isotype	Antigenic Protein (kDa) ^a^	50 Fish Species		3 Shellfish Species		11 Land Animal Species		4 Food Additives	
			Positive	Negative	Positive	Negative	Positive	Negative	Positive	Negative
8F5	IgG2a	36	48	2 (swordfish and yellowfin tuna)	0	3	0	11	0	4
2A4	IgG1	36	50	0	0	3	11	0	0	4
3F5	IgG2b	36	50	0	0	3	9	2	0	4

^a^ The antigenic proteins were determined by western blot in cooked (100 °C, 8 min) fish extracts of eight representative fish species.

**Table 3:** Changes in soluble protein concentrations of fish samples (yellowfin tuna, swordfish, cod) with different heating times (0–15 min).

Heating Time	Yellowfin Tuna		Swordfish		Cod	
	Concentration (mg/mL) ^a^	Ratio of Concentration (%)	Concentration (mg/mL) ^a^	Ratio of Concentration (%)	Concentration (mg/mL) ^a^	Ratio of Concentration (%)
0 min	16.09 ± 0.64	100	8.73 ± 0.23	100	3.83 ± 0.09	100
1 min	12.67 ± 0.13	78.7	7.69 ± 0.12	88.1	3.72 ± 0.08	97.1
3 min	6.00 ± 0.11	37.3	3.70 ± 0.06	42.4	1.78 ± 0.04	46.4
5 min	0.16 ± 0.02	1	0.30 ± 0.03	3.4	1.09 ± 0.04	28.5
10 min	0.17 ± 0.01	1.1	0.27 ± 0.01	3.1	1.04 ± 0.03	27.2
15 min	0.29 ± 0.02	1.8	0.31 ± 0.01	3.6	1.17 ± 0.01	30.5

^a^ The protein concentration was determined in triplicate and presented as mean ± standard division.

## Data Availability

Not applicable.

## Supplementary Materials

The following are available online at https://www.mdpi.com/article/10.3390/foods10102360/s1, Figure S1: Western blot analysis of cooked fish samples using mAb 2A4 (**a**) and mAb 3F5 (**b**) supernatant (1:5), respectively.
