# Supplementary material for: Development and Characterization of Monoclonal Antibodies for the Detection of Fish Protein
Source: Foods. 2021 Oct 4;10(10):2360. doi: 10.3390/foods10102360 (PMC8535609; doi:10.3390/foods10102360)
Supplement: Supplementary file 1 [file foods-10-02360-s001.zip › foods-1346117-supplementary.pdf]

(a)

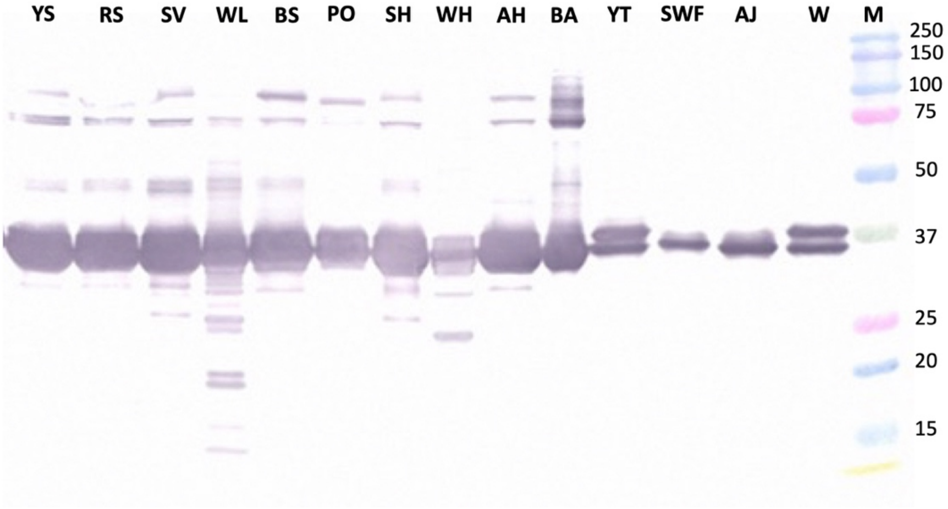

(b)

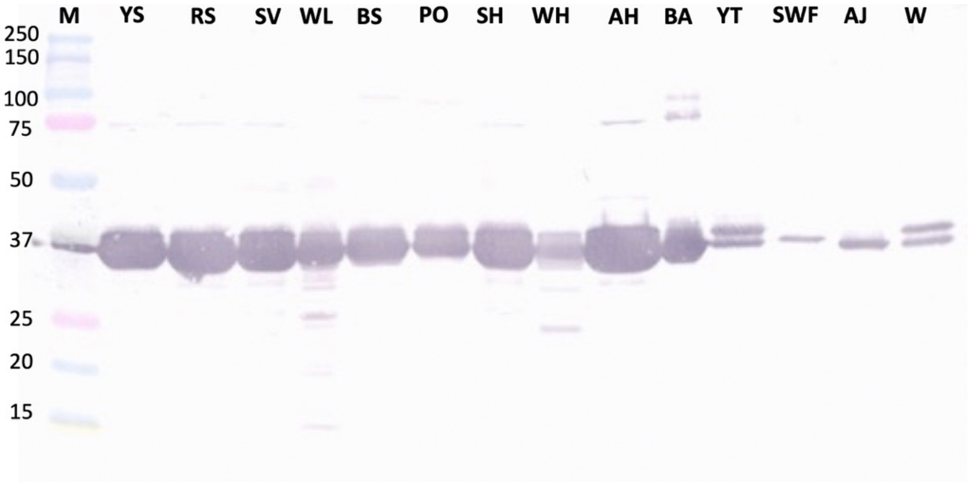

Figure S1. Western blot analysis of cooked fish samples using mAb 2A4 (**a**) and mAb 3F5 (**b**) supernatant (1:5), respectively. The amount of sample loaded on the 12% SDS-PAGE was 6 μL per lane. M: molecular weight marker. The abbreviations are as below. YS: Yellowtail snapper; RS: red snapper; SV: vermilion snapper; WL: wavy lined grouper; BS: black sea bass; PO: pollock; SH: sheephead; WH: whiting; AH: Alaskan halibut; BA: basa; YT: yellowfin tuna; SWF: swordfish; AJ: amberjack; W: whaoo
